# Developing a roadmap to reach and sustain 90% full immunization coverage through a cross-sectoral system strengthening strategy in Bihar, India

**DOI:** 10.1186/s12913-024-11380-7

**Published:** 2024-08-14

**Authors:** Manish Ratna, Sanjay Kumar Singh, Narendra Kumar Sinha, Mandar Kannure, Mehak Bhatia, Mahesh Kumar Aggarwal, Suresh Kumar Dalpath, Saurabh Sharma, Vama Shah

**Affiliations:** 1 William J. Clinton Foundation, New Delhi, India; 2 State Health Society, Government of Bihar, Patna, India

**Keywords:** Routine immunization, Public health, Strategic planning, Health system strengthening, Intervention, Immunization coverage

## Abstract

**Introduction:**

Reducing childhood mortality by curtailing the incidence of vaccine preventable diseases is contingent upon a robust and high-performing routine immunization system. According to the available data, the full immunization coverage (FIC) in the state of Bihar (India) has reached ~ 71%. While the government aspires to reach 90% FIC, a systematic evidence-based investigation of the reasons behind underimmunization as well as the identification of drivers and enablers to reach and sustain 90% FIC is critical. This study aimed to review the factors leading to underimmunized children in the state of Bihar and develop a forward-looking roadmap to reach and sustain 90% FIC by adopting a system strengthening approach.

**Method:**

We conducted a desk review, followed by extensive stakeholder interviews and field visits to document and analyze the data and evidence relevant to routine immunization system performance in the state of Bihar. The stakeholders included the State Immunization Officer, District Immunization Officers, Block-level health officials, representatives from development agencies, healthcare workers, and caregivers. A total of eighty-six structured interviews were conducted, which included qualitative and quantitative parameters.

**Result:**

While positive results were observed from the assessment of Bihar’s immunization system, the implementation of targeted strategies for supply, service delivery and demand can provide a means to achieve FIC of 90%. The roadmap developed by the Government of Bihar enlists 40 + interventions across key thematic areas and has been prioritized over a 5-year time horizon as short, medium, and long-term milestones to achieve 90% FIC. These interventions include strengthening the data availability and quality, improving the governance and review mechanism, augmenting the capacity of health workers involve with immunization programme, and initiatives to increase demand for immunization services.

**Conclusion:**

The Bihar’s Immunization Roadmap development project work follows a methodical approach to assess and identify intervention to improve immunization coverage and can provide information and reference to other states and countries that are aiming to formulate similar action plans.

**Supplementary Information:**

The online version contains supplementary material available at 10.1186/s12913-024-11380-7.

## Background

Immunization is a key component of primary health care and is critical for preventing and controlling infectious disease outbreaks. It averts 3.5–5 million deaths every year from diseases such as diphtheria, tetanus, pertussis, influenza, and measles [1, 2]. Between 2010 and 2018, 23 million deaths were averted due to the measles vaccine alone [3]. Despite an increased focus on immunizing children, which helped increase global immunization coverage to 85% in 2019, 19.7 million children remained vulnerable to vaccine-preventable diseases [4]. Almost a quarter of such children, around ~ 5 million, reside in India. Although India’s Full Immunization Coverage (FIC) among children aged 12–23 months, increased from 35.0% in 1992–93 to 76.4% in 2019–21 [5, 6], achieving 90% FIC remains a challenge. Highly populous Indian states such as Bihar and Uttar Pradesh exaggerate the challenge [7].

**Fig. 1:**
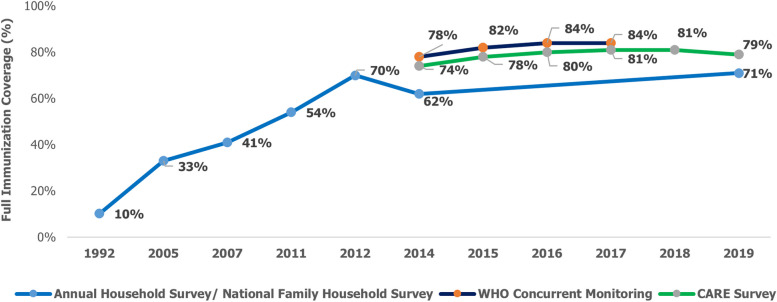
Full immunization coverage in Bihar

Bihar with a population of ~ 120 million people is the third most populous state in India and home to 9% of India’s resident population [8]. The state has an annual birth cohort of 3 million, which is an 11% share of the national cohort. Bihar’s FIC in 1992–93 was 10.2% in 1992–93, which increased to 71.0% by 2019–20. The FIC increase contributed towards a reduction in Bihar’s Infant Mortality Rate from 42 in 2015 to 29 in 2019 [9]. Despite the rapid escalation of FIC between 1992 and 2019, a gradual plateauing of coverage after 2014–15 can be observed (refer Fig. 1). This necessitated a comprehensive assessment and a need for developing a newer set of interventions that will help drive FIC to 90% and beyond.

The Government of Bihar (GoB) partnered with the William J. Clinton Foundation (WJCF) to develop an understanding of the program and adopted an evidence-based approach to construct a multiyear roadmap to help achieve UIP goals. The roadmap describes more than forty high-impact and innovative interventions that GoB can undertake over short-, medium- and long-term time horizons to strengthen immunization systems and achieve 90% FIC in Bihar.

### Introduction to universal immunization program and the need for a coverage improvement plan in Bihar

The Universal Immunization Program (UIP) of India is the largest of its kind in the world, serving an annual cohort of 26 million infants and 29 million pregnant women through 12 million annual sessions [10]. The UIP was launched in 1985, and over the years, has evolved through the new vaccine introductions, self-sufficiency in in-country vaccine production, inclusion of an open vial policy, upgrades in the cold chain, vaccine and logistics management, improvements in the disease surveillance mechanism, and management of adverse events following immunization [11, 12].

A strong momentum exists in India towards RI strengthening, as evidenced by multiple initiatives and policies. [5, 6, 13–15] The launch of Mission Indradhanush in 2014 and its intensified version (IMI) subsequently, alongwith a continuous monitoring of the program, highlight the government's targeted efforts to improve FIC equitably [16–18]. The incorporation of new vaccines such as the Rotavirus vaccine, Pneumococcal Conjugate Vaccine, and Measles-Rubella vaccine into the UIP demonstrates an expansion of the RI program to cover more diseases, reflecting a commitment to improving public health outcomes [7, 19–22]. Additionally, digital platforms such as the Electronic Vaccine Intelligence Network (eVIN) have been implemented to improve vaccine logistics and stock management, enhancing the efficiency and reliability of immunization services [23, 24]. The collaboration with international organizations such as GAVI, UNICEF, and WHO further strengthens the program through technical and financial support [25]. Public awareness campaigns and policy frameworks, such as the National Vaccine Policy (2011) and Comprehensive Multiyear Plan (2018–2022), also underscore the strategic efforts to ensure equitable access to RI services across the country [26–28]. Ministry of Health and Family Welfare (MoHFW), recognizing the need to catalyze FIC improvement in the country, through improved routine immunization (RI) planning and strengthened health systems, released a roadmap for achieving 90% FIC to the states/UTs [5], which recommended the states to develop Immunization Coverage Improvement Plan for districts with < 90% FIC. Additionally, state of Bihar also has launched several high-impact coverage improvement interventions, including *Muskan Ek Abhiyan*, *Mukhyamantri Saghan Tikakaran Abhiyan, and Mukhya Mantri Kanya Utthan Yojana* [29–32]. These initiatives collectively indicate a strong political will to achieve high FIC and equity.

Not restricting to Bihar as a geography, the authors found limited evidence pertaining to long-term strategic planning conducted for increasing immunization coverage and health system strengthening. Strategic frameworks, such as Immunization Agenda 2030 [1] and the Global Routine Immunization Strategies and Practices (GRISP) initiative by WHO [33], provide helpful inroads to planning for coverage and equity improvement. However, such frameworks lack the granular, context-specific detail necessary to address the unique challenges and opportunities Bihar faces. This necessitated the development of a comprehensive and actionable roadmap, informed by the local context and challenges, which could support the immunization stakeholders in Bihar in achieving and sustaining 90% FIC.

**Fig. 2:**
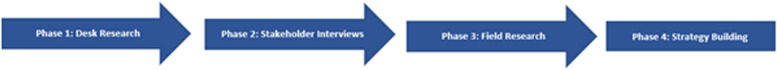
Phases in the study design

This paper intends to identify barriers and challenges toward 90% FIC in Bihar, and best practices and innovations unique to Bihar. This paper also uncovers pathways, taking a system-strengthening approach, to achieve 90% FIC while exploring the sustainability and scalability perspectives. The next sections cover the data collection and analysis methods, followed by results, discussion on key thematic areas for FIC improvement and sustainability (Phases in the study design are outlined in Fig. 2).

## Methods

### Desk review

The first phase of the exercise involved a desk review, utilizing the published literature and available datasets (referred in Table 1) to derive insights related to the Routine Immunization Program in Bihar as well as to define the line of questioning for the stakeholder interviews and field research. The desk review was conducted between July and September 2020.

**Table 1:** Components included in the desk review

S. No	Desk Review Components	Data Sources
1	Published Literature	Peer-reviewed research papers, policy papers, publications from government and key agencies, and media reports
2	Quantitative Data Analysis	National and Subnational Surveys/Administrative data on Health and Immunization (Annual Household Survey, Coverage Evaluation Surveys, HMIS, RHS, NHM-PIP, NFHS, and Sample Registration System)

### Stakeholder interviews & on-site session observations

In the second phase of the exercise, the objective was to understand the perspectives of the interviewees (Table 2) on the challenges and opportunities for improvement across program components. The interviews were conducted over a month, from October to November 2020, through a combination of in-person interviews, digital interactions and on-ground conversations and observations through visits to health facilities and session sites. The interviews lasted between 30 and 90 min, during which open-ended questions on components related to Bihar’s Routine Immunization program were asked, enabled by a set of quantitative and qualitative questions (see Additional file 2). These findings were complemented with on-ground assessments via field visits to immunization session sites across five districts of Bihar out of the fourteen districts included in the study (Table 3). The on-ground assessments were necessary due to multiple factors, such as reducing bias in the collected data from interviewees in the second phase; uncovering hidden insights and opportunities that stakeholders may not be aware of or able to articulate in interviews; and developing a deeper understanding of beneficiaries’ and healthcare workers’ needs and behaviors, which might be helpful in designing solutions that are more tailored and documenting some of the implemented best practices. This was supplemented with a round of telephone interviews with district immunization officers from nine districts. This phase provided for the qualitative synthesis of insights collated and helped ascertain factors that explain Bihar’s success in routine immunization over the past two decades and identify persistent challenges.

**Table 2:** List of organizations and interviewees and the number of interviews conducted in the study

Organization/Affiliation	Interviewee	Total
Government of Bihar/State Health Society/the office of the state immunization officer	• Block Accounts Managers (BAM) • Block Community Managers (BCM) • Block Health Managers (BHM) • Data Entry Operators (DEO) • District Immunization Officers (DIO) • Medical Officers In-Charge (MOIC) • State Cold Chain Consultant (SCCC) • State Immunization Officer (SEPIO)	18
Front Line Workers	• Auxiliary Nurse Midwives (ANM) • Accredited Social Health Activists (ASHA) • ASHA Facilitators • Anganwadi Workers (AWWs) • General Nurse Midwives (GNM) • Vaccine and Cold Chain Handlers (VCCH)	17
Session Site Observations and Facility Visits	• Cold Chain Points • District and Sub District Hospitals • Immunization Session Sites • Model Immunization Centers • Primary Health Centers	13
Community Representatives	• Caregivers present at Session Sites • Community members	20
Development Agencies	• Bill and Melinda Gates Foundation (BMGF) • Piramal Foundation • Population Foundation of India (PFI) • Program for Appropriate Technology in Health (PATH) • United Nations Development Programme (UNDP) • United Nations Children’s Fund (UNICEF) • William J. Clinton Foundation (WJCF) • World Health Organization (WHO)	18
**TOTAL**		**86**

**Table 3:** List of districts in Bihar covered during the study

S. No	Districts	Mode of Data Collection
1	East Champaran	Field Visit
2	Jehanabad	Field Visit
3	Muzaffarpur	Field Visit
4	Nalanda	Field Visit
5	Patna	Field Visit
6	Bhagalpur	Telephonic Interview
7	Gaya	Telephonic Interview
8	Gopalganj	Telephonic Interview
9	Kishanganj	Telephonic Interview
10	Lakhisarai	Telephonic Interview
11	Madhubani	Telephonic Interview
12	Saran	Telephonic Interview
13	Supaul	Telephonic Interview
14	Vaishali	Telephonic Interview

The study was conducted with a sample size of eighty-six interviewees. Purposive sampling was adopted to identify the interviewees; however, to ensure representativeness across the immunization programme, key segments were identified, and interviews were conducted until data saturation was achieved. The inclusion criteria for the interviewees were as follows:

- Respondents representing all levels of the immunization system – block, district, and state levels.
- Respondents representing a range of organizations/affiliations, including the Government of Bihar, the office of the state immunization officer, development agencies, frontline workers, and community representatives. This heterogeneity ensured varied inputs and data, the details of which are presented in Table 2.

The interactions with stakeholders, conducted in person and virtually, were first documented individually on MS Word and subsequently collated with the responses from other interviews on MS Excel. The responses from these interviews were then deductively coded (manually) via qualitative data analysis software (NVivo's Version 12) into themes using a hierarchical coding frame that was congruent with the categorizations used during the desk review phase to maintain the structural integrity and consistency of the analysis. After this step, the authors synthesized the coded information to arrive at key insights across programmatic components.

### Roadmap development

The third phase focused on converting the in-depth assessment of the collected data and insights into strategies, identifying key areas for action, and assessing the feasibility and potential impact of the interventions. This activity was conducted in November and December 2020. These thematic areas included healthcare infrastructure; availability, effectiveness, and supervision of healthcare workers; governance, review systems and finance; immunization service delivery; demand generation for immunization; vaccine availability, shortage, and logistics; and immunization data systems.

### Maturity index

The maturity model is a conceptual framework that consists of a sequence of discrete maturity levels for a class of processes in one or more business domains and represents an anticipated, desired, or typical evolutionary path for these processes [34]. Before describing the interventions and associated implementation scale used in this study, the Maturity Index was conceptualized, in line with the Maturity Model, as a mechanism for subjectively assessing the level of maturity or readiness of identified program components based on criteria such as policies, procedures, training, infrastructure, and governance. Each thematic component and subcomponent were assigned a maturity index for the current level and the target level. The current level was collectively articulated using the observations and findings from the previous two phases, while the target level was assigned by considering the timeline of the roadmap and the feasibility of achieving the intended impact through potential interventions. The maturity index was designed to have a four-point scale as follows: (1) basic, (2) developing, (3) advanced, and (4) leading. The qualification parameters under the scale were uniquely defined for each program component and subcomponent, comprehensively covering the possible range of progress levels under the program component/subcomponent. The target maturity level indicated against each component/subcomponent emerged as a variable, dependent on the timeline identified under the roadmap’s purview as well as the feasibility of the intended achievement. After the index was assigned to the program component/subcomponent, a basket of potential interventions was combined through a process of research, consultation, and brainstorming.

The interventions were then arranged into short (< 6 months), medium (6 months to 2 years) and long (2 to 5 years) time horizons based on the need for a proof of concept, the time needed for scale-up, and dependencies on other interventions. Finally, for each intervention, a specific mechanism of action was identified for the design, implementation, and institutionalization phases, along with the recommended scale of intervention.

## Results

The insights from the desk review, stakeholder interviews and field visits were analyzed and segmented across the priority thematic areas, as presented in this section.

### Healthcare infrastructure

Data from the RHS [8] show that a typical health facility in Bihar, at every administrative level, caters to a much greater population load than recommended by the Indian Public Health Standards [35] (Table 4). As of March 2019, Subhealth Centers and Primary Health Centers (rural) were serving ~ 2x times population, while Primary Health Centers (urban) and Community Health Centres (rural) served ~ 3x and ~ 6x population respectively, against the IPHS benchmark. Access to health facilities continues to remain a challenge since there has been a slower addition of new health facilities in Bihar between 2007–2019 (Table 5).

**Table 4:** Facility wise average population load in Bihar

Facility Type	Geography	Population Load per Facility (March 2019)	IPHS Benchmark
Sub-Health Centre	Rural	10,875	5,000
Primary Health Centre	Rural	56,974	30,000
Primary Health Centre	Urban	145,014	50,000
Community Health Centre	Rural	721,285	120,000

**Table 5:** Number of health facilities in Bihar

Health Facility Type	Number of Health Facilities in Bihar (as per RHS 2020–21)				
	**2007**	**2012**	**2017**	**2018**	**2019**
**Sub-Health Centers**	8,909	9,696	9,949	9,949	9,949
**Community Health Centers**	70	70	150	150	150
**Primary Health Centers and Additional Primary Health Centers**	1,648	1,863	1,899	1,899	1,899

### Availability, effectiveness, and supervision of healthcare workers

**Fig. 3:**
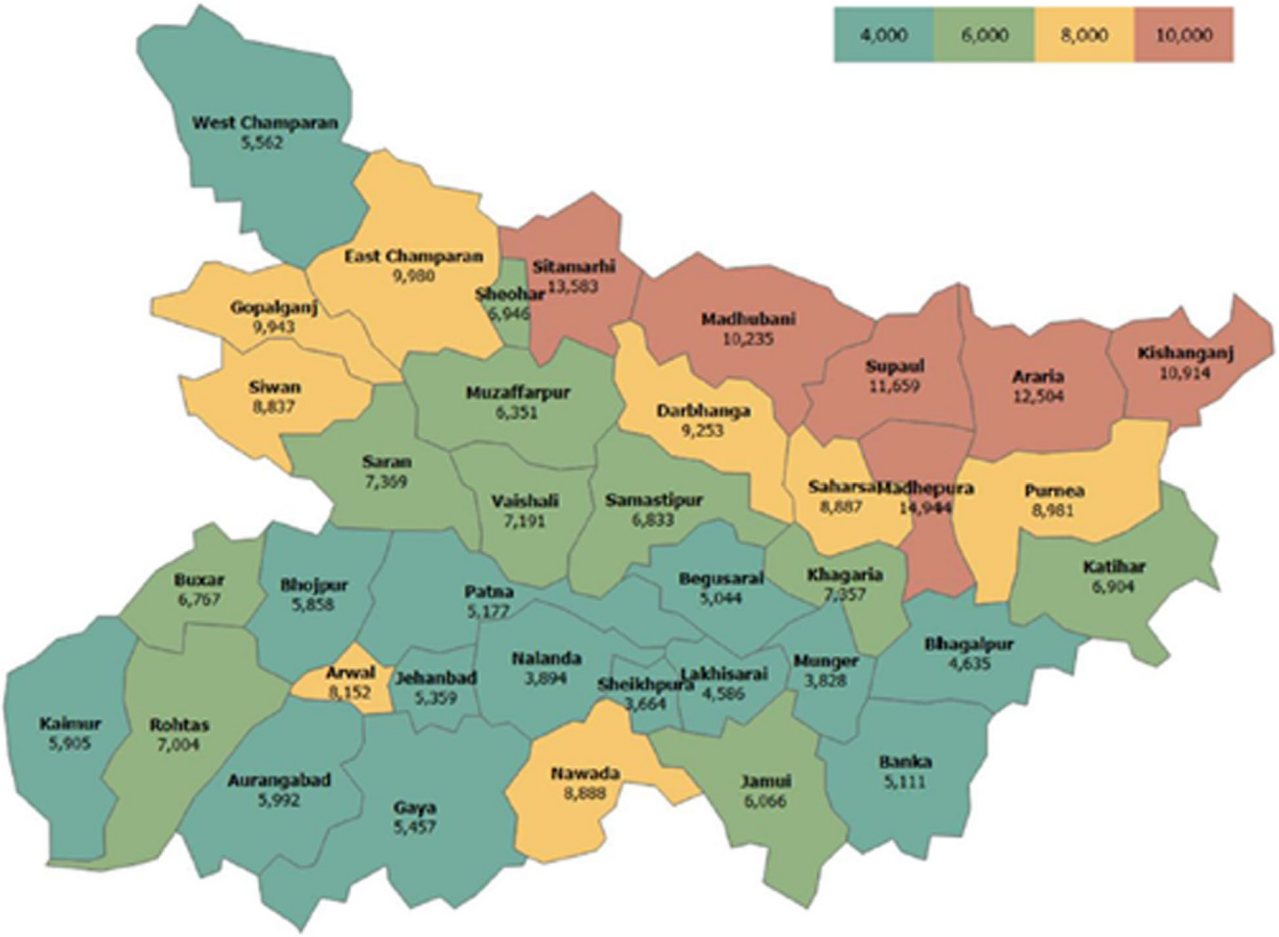
Population dependence per ANM across districts in Bihar (2018–2019)

In Bihar, ANMs are the primary vaccinators, while ASHA workers are the mobilizers. As per RHS 2020–21, Bihar had 20,403 ANMs in position against the requirement of 12,190 ANMs at SHCs and PHCs in rural areas [36]. At the district level, however, it was observed that there was a significant additional load on ANMs in certain northern districts, such as Araria, Madhepura, Madhubani, Kishanganj, Sitamarhi and Supaul, where the average population mapped to an ANM was ~ 1.5–2.5x the ideal population load of 5,000 per ANM (refer Fig. 3) [8].

The supportive supervisory cadre provides handholding support to the ANMs at sessions, monitors the quality of sessions and ensures adherence to UIP guidelines. They also supervise cold chain stores and conduct house-to-house visits to identify left-out or dropout children. The number of supervisors is insufficient, as indicated by the high number of vacancies, which impacts session quality and adherence to UIP guidelines due to subpar supervision [36, 38]. Supervision in the Immunization Program is currently being provided by development agencies, Block Health Managers (BHM) and Block Community Managers (BCM). However, as per the interviewees, the involvement of BHMs and BCMs in supervision activities is limited and variable due to the variety of tasks they are supposed to perform and the programs for which they are accountable.

Apart from availability, the lack of skilled health workers is another key challenge in Bihar [36, 38]. While multiple mechanisms for capacity building exist, studies show that coverage of immunization training is lower than required for medical officers (MOs), ANMs and ASHAs. Interviewees mentioned that topics such as data recording and reporting processes, immunization waste management, and delivery of the four key messages to beneficiaries are where ANM training needs reinforcement. Currently, capacity building of frontline workers is done through multiple mechanisms, such as two-day immunization health worker module training for ANMs, orientation training for newly hired or deployed ANMs, and training for new vaccine introduction. Needs-based upskilling is conducted during weekly ANM meetings. 60% and 94% of ASHAs were in position against the target for urban and rural areas, respectively, in Bihar in 2019 [39]. Additionally, a relatively lower proportion of ASHAs were trained against the target compared to other states, as per the Annual ASHA Update 2020–21 [39]. Interviewees mentioned that ASHAs in Bihar require capacity building on topics such as mobilization, due lists, headcount surveys, and documentation practices.

### Governance, review systems and finance

The immunization program is managed by the Health Department under the leadership of the State Immunization Officer, with district and block-level implementation managed by DIOs and MOICs, respectively. Program reviews involve mechanisms such as State and District Task Force for Immunization (STFI & DTFI), Weekly Reviews at District and Block level (DWR & BWR). However, Bihar’s UIP Review (2018) indicated that only 63% of the planned DTFI meetings were held, and stakeholder interviewees cited that improvements are needed in regularizing and enhancing quality of these reviews. Field visits revealed that DWR and BWR agendas focused on development agency’s supervisory visit findings, indicating an opportunity to better use program data for decision-making. Limited participation of MOs in supervision and reviews was noted. High budget utilization was for mobilizers (ASHA) and porter (Alternate Vaccine Delivery) incentives. However, there is a need to improve awareness of the available funds and processes to avail them.

Stakeholders mentioned that that the ASHAs are a key health workforce and to strengthen this part of the program, GoB has revised their incentive structure for RI. ASHAs now receive a minimum of INR 50 (for atleast 5 beneficiaries mobilized), with additional INR 10 per extra beneficiary mobilized, and a maximum earning potential of INR 350 per session (for atleast 35 beneficiaries mobilized).

### Immunization service delivery

**Fig. 4:**
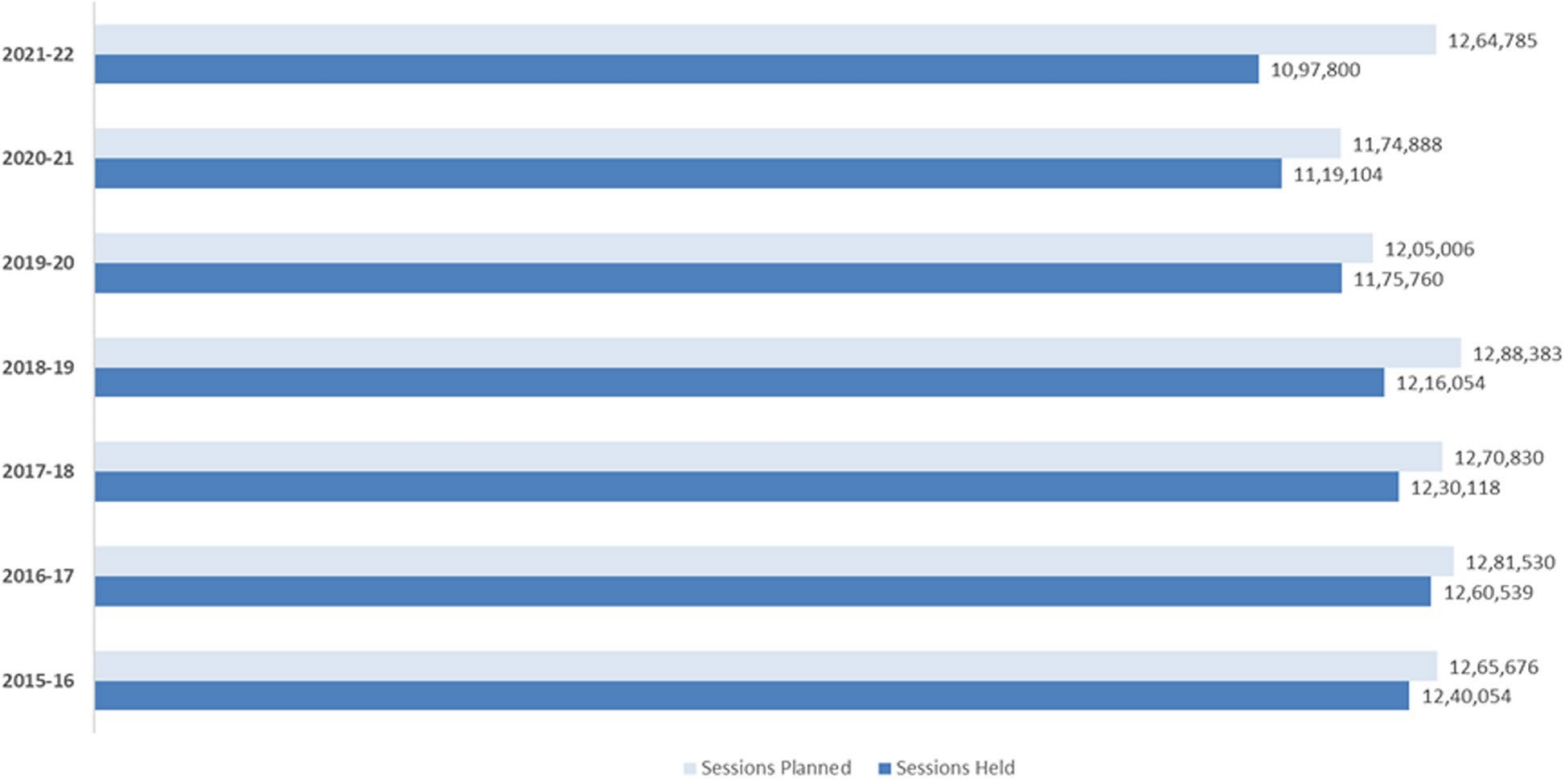
Planned and held immunization sessions in Bihar

 ~ 1.2–1.3 million immunization sessions are planned each year in Bihar, of which up to 98% are held (refer Fig. 4) [40]. However, some of the northern districts in the state underperform on this indicator, partially attributable to the flood-led challenges. The total number of sessions planned, and sessions held in Bihar have decreased consistently in the recent past.

Immunization microplanning is the basis for the delivery of immunization services to a community. The availability of updated and complete microplans directly affects the quality of services provided. Microplans are prepared for a one-year period but must be reviewed every quarter and updated every six months. Interviewees mentioned that while microplans are developed annually, they are not updated biannually as needed. This may lead to missed areas and beneficiaries. At the state level, interviewees indicated that increasing the number of session sites is required to ensure better access to communities, while rationalization is required in such geographies, where the frequency of holding immunization sessions is too high or lower than needed.

Interviewed District Immunization Officers (DIO) mentioned that migration is prevalent in their districts, which impacts the program’s performance. While in-migration potentially leads to a greater workload for ASHAs and ANMs, out-migration may impede them from ensuring that follow-up is administered to beneficiaries. Institutionalizing the beneficiary tracking and tracing mechanism has emerged as a key requirement for ensuring the immunization of migrant and underserved populations.

### Vaccine availability, shortages, and logistics

**Fig. 5:**
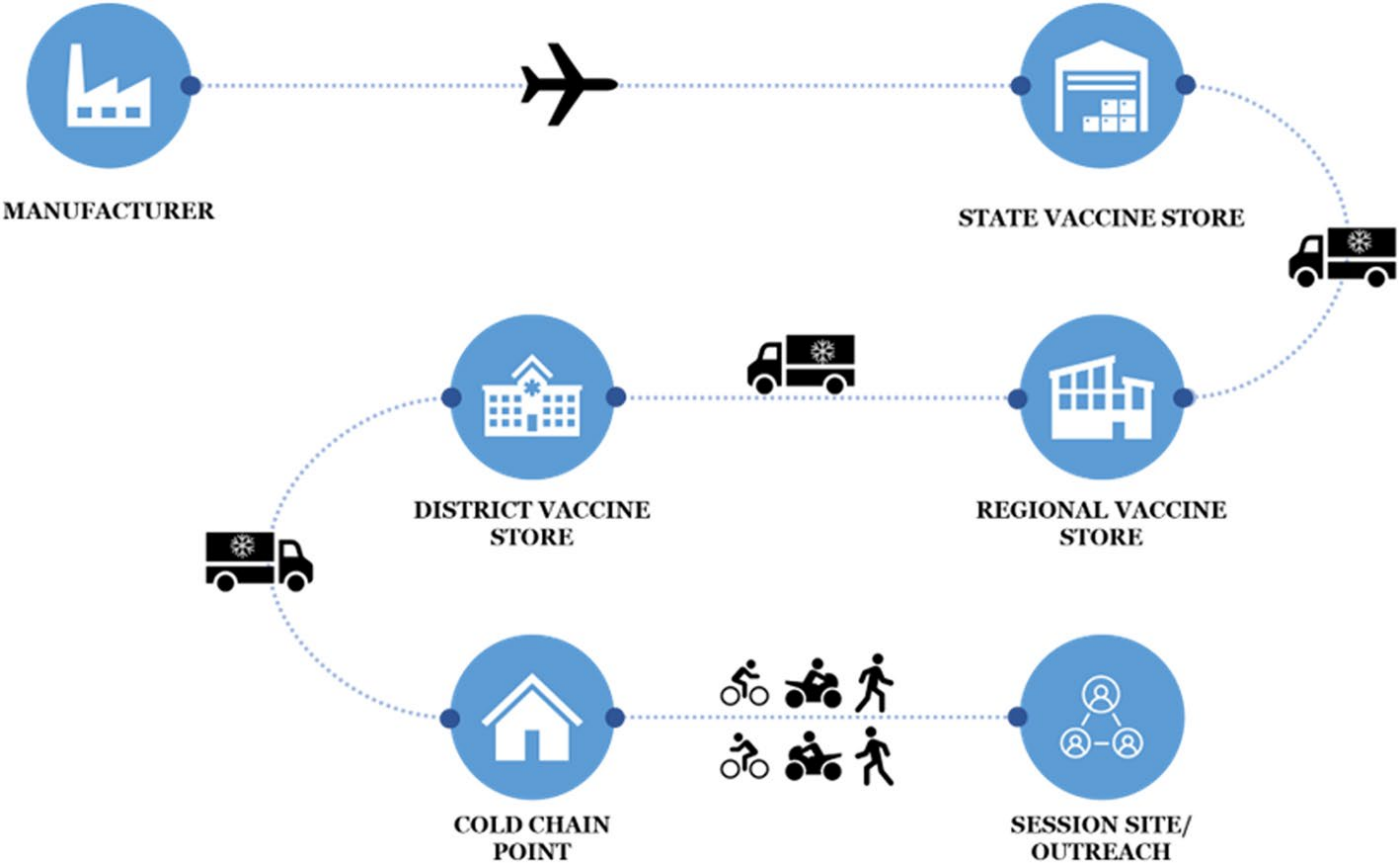
Bihar's immunization supply chain

The immunization supply chain (iSC) is the backbone of a well-performing immunization program. Bihar’s iSC network consists of four tiers, namely, a state vaccine store (SVS), a regional vaccine store (RVS), a district vaccine store (DVS), and cold chain points (refer Fig. 5). Cold chain points are typically located in government health facilities such as Community Health Centers and Primary Health Centers. The vaccine supply and cold chain inventory are managed through the electronic Vaccine Intelligence Network (eVIN) and the National Cold Chain Management Information System (NCCMIS).

The iSC in Bihar has benefited from the national- and state-level initiatives and has evolved in terms of infrastructure, people, systems, and processes. In 2011, Bihar had eight RVSs, 11 DVSs and 160 CCPs [41]; however, in 2017, it had one SVS, 9 RVSs, 38 DVSs and 606 CCPs [42] as part of its cold chain network, which has further expanded since then. The number of DVSs has more than tripled, while the number of CCPs has almost quadrupled.

In Bihar, most blocks have one CCP, which acts as the hub for vaccine distribution to all the session sites in the block. Administrative data indicated that, on average, Bihar had a relatively greater number of session sites attached to the CCP than did other states [43]. Interviewees mentioned that multiple session sites in their block were under one hour away from the CCP, but to improve access at the hard-to-reach session sites, there was a need to open new CCPs that are geographically appropriate. However, opening a new cold chain point may lead to feasibility problems such as a lack of equipped buildings, limited electricity supply and trained HR shortages. Interviewees mentioned that the availability of vaccines at CCPs as well as session sites has improved over the last ten years, and the number of stockout instances has decreased.

All the cold chain technicians (CCT), who are in position, were trained in the maintenance and repair of cold chain equipment, but there were a limited number of CCTs available for managing the repair instances. Additionally, the earmarked outlays for the repair and maintenance of CCPs are at the rate of ~ Rs. 1,000 per CCP instead of being on a per equipment basis (as per Bihar’s NHM PIPs & ROPs in 2020–21 [37]), the increase in cold chain equipment per CCP due to recent procurements and relatively slower addition of new CCPs in Bihar has put pressure on the budget outlay.

VCCHs, responsible for managing the vaccine supply chain, were found to be available at all the visited CCPs and trained. However, on multiple instances, the VCCH was found to have multiple responsibilities at the health facility. The average population load per CCP is higher in Bihar compared to the GoI benchmark [43], with some of the visited CCPs mapped to ~ 2x the recommended population size. This led to many VCCHs spending more time managing the vaccine distribution than ideal and pulling them away from other competing priorities for which they are responsible, leading to probable subpar service delivery at the health facility.

The observations from the visited session sites indicated that all the essential vaccines, syringes, and diluents were available. However, there was insufficient availability of vitamin A syrup, biomedical waste bags, and hub cutters.

### Immunization data system strengthening

Bihar has adopted all the nationally implemented immunization data systems, such as the HMIS, the Reproductive and Child Health (RCH) portal, the eVIN, the NCCMIS, and the Surveillance and Action for Events Following Vaccination (SAFE-VAC). Interviewees claimed that the data recording and reporting processes in Bihar are strong, but there were improvement opportunities in the accuracy, consistency, and completeness of HMIS’s immunization data, which is one of the key databases.

Interviewees mentioned the headcount survey’s supervision to be a challenge and often found its quality to be subpar. At the session sites, it was observed that all beneficiaries listed in the headcount survey were also present in the due list; however, additional beneficiaries not listed in the headcount survey were found. The tally sheets, filled at the end of each session, were often found inconsistent with the HMIS-MPR (Monthly Progress Reports) filled at the end of every month. As per the interviewees, the data validation process involving MPRs and tally sheets needed improvement, as these are the key sources of information and are referred to during the program reviews.

Multiple DIOs cited that DEO’s capacity to understand the definition of FIC, analyze HMIS data, and identify discrepancies across reports was limited, which might be aggravated by the high workload and multiple ad hoc reporting work. Also, the adoption of the RCH portal [44], which was designed for the early identification and tracking of beneficiaries throughout the service delivery lifecycle, is relatively lower in Bihar than in other states, driven by the limited use of portal data for immunization-related decision making.

The interviewees mentioned that the adverse events following immunization (AEFI) surveillance system is useful at all levels but is partially meeting its objective due to underreporting. Severe and serious AEFI cases are generally reported on-time through the Case Reporting Format, and AEFI Causality Assessment Meetings are held to review the AEFI cases; however, there are challenges related to the reporting of minor AEFI cases.

### Demand generation for immunization services

Healthcare-seeking behavior for immunization across population groups in Bihar falls on the side of the public sector, with only 1.4% of rural children being immunized at private facilities [31]. Key challenges include an awareness gap among caregivers (39%), AEFI apprehension (27%), and other reasons, such as an operational gap (9%), refusal (6%) and child travelling (16%) (as per the MoHFW’s Annual Immunization Dashboard 2020). Additionally, the demographic context of the state also indicates a prevalence of seasonal migration, especially during traditional festivals such as *Chatth, Durga Puja*, and *Holi*. With a proclivity to miss documenting the migratory population during headcount surveys and microplanning exercise, migrants are often the contributors to cases of delayed and sometimes missed vaccination.

Multiple districtwide and statewide innovations have been implemented to increase the demand for immunization. The *Mukhyamantri Kanya Utthan Yojna* (a state government-led policy aimed at the uplift of girls) is a flagship demand-side intervention implemented by the Government of Bihar, under which Rs. Two thousand is payable on the immunization of girl children under the age of 2 years [32]. Conversations with block officials indicated that even though beneficiaries are aware of this scheme and grassroot-level health workers (ASHAs and ANMs) have been trained to fill incentive disbursement forms, beneficiaries are sometimes unable to receive these benefits due to the unavailability of Aadhaar Cards. Other initiatives, as mentioned by interviewees, include the *Aapki Sarkar Aapke Dwar (*Your Government at Your Doorstep initiative) drive, the establishment of *Model Immunization Centers* and the pursuit of interdepartmental collaboration with the ICDS and JEEViKA self-help groups (Bihar Rural Livelihoods Promotion Society) to generate awareness and demand for immunization services.

Interactions with interviewees indicated that while the vaccine hesitancy and refusal cases might be minimal overall, there exist some areas and communities in Bihar where context specific demand generation work is needed. These communities typically have a population that has historically faced social exclusion and vulnerabilities which disproportionately impact their access to immunization services and inequity. Community members from such population cohorts need targeted visits by health workers or sometimes even by senior government officials to convince them to take their children for immunization. To mobilize such communities, *Vikas Mitras* acts as the interface between health workers and their community to spread information about public health among other government services. As they belong to the same community as beneficiaries, there is an increased sense of trust among beneficiaries. Such examples highlight the complex and intertwined role of social, cultural, and economic factors in the demand for and uptake of immunization and vaccination services.

Conversations with caregivers and beneficiaries indicated that their awareness of the technical aspects of immunization services is low and that they are usually dependent on their ASHAs for much of the informational support, including the next service delivery date. The incidence of AEFIs tends to influence caregivers against continuing with the immunization schedule and potentially dropping out. Paracetamol was being given at most of the sessions; however, mostly in the form of tablets, as there was a shortage of Paracetamol syrup. When asked if they would immunize a sick child, caregivers responded by either saying that they would not immunize or that they would consult the ANM and ASHA before deciding, which indicates trust in the health workers. As per a DIO, dropout occurs primarily for two reasons: the child being sick or traveling. A combination of factors, including but not limited to these two reasons, can lead to supply–demand gaps.

## Discussion

### Increasing healthcare staff and infrastructure

The present study sheds light on the relative shortage of health infrastructure in Bihar, emphasized through high population loads per facility, to accessibility challenges. Health infrastructure and staff capacity need to be augmented to match the needs of the population across the state. More SHCs are needed to improve access to immunization services at the last mile, as ANMs can be hired and positioned there, which will both reduce the population burden per ANM and improve outreach immunization service delivery to underserved communities. New CCPs should be identified in areas with hard-to-reach session sites and operationalized in mission mode. Overcoming this shortage should help eliminate health worker- and infrastructure-related gaps.

Since the development and launch of the roadmap, there has been a significant increase in the number of CHC, PHC, APHC and SHC patients. The number of CHCs, PHCs, APHCs, and SHCs increased from 150 to 250 (+ 67%), 1899 to 1,932 (+ 2%), and 9,949 to 10,258 (+ 3%), respectively, between the 2020–21 (RHS statistics) and 2022–23 (State Health Society Bihar’s administrative data).

As an innovation aimed at alleviating the problem of the paucity of sufficient health facilities in Bihar, the government innovated the concept of *Model Immunization Centers,* where high-quality immunization services are provided at select health facilities [45]. This has led to measurable positive changes. In addition, *Model Immunization Corners* are being developed at public hospitals to motivate caregivers to seek immunization services at public health facilities [46]. Consistent with the findings in the literature review, Model Immunization Centers and Model immunization corners have been observed to provide high-quality immunization services in a comfortable environment to caregivers and beneficiaries [45]. These are popular platforms for increasing coverage, especially in urban areas, and building community confidence in the quality of immunization services. As of May 2022, Bihar’s Health Department has 19 Model Immunization Centers and 154 Model Immunization Corners operational, with more such centers in the pipeline.

### Healthcare workers’ competencies and effectiveness

The availability of sufficient ANMs and their rational distribution would ensure a reasonable workload on each ANM, thereby allowing ANMs to focus on delivering quality immunization. In the past few years, the government has taken proactive steps toward augmenting the number of ANMs in position through new hiring as well as existing workforce rationalization, which has recently increased ANM availability across the state [36, 38]. However, the session load per ANM is still high in multiple districts and blocks. In practice, this has translated to the ANMs conducting up to four sessions per week in such blocks and districts, against the ideal workload of two sessions per week.

In addition to the availability problem, a lack of skilled healthcare workers also came up as a challenge. The focus must be placed on both the availability of healthcare workers and the skill building and mentorship of healthcare workers. After an initial capacity building needs assessment of the existing health workers, a plan to systematically upscale their capacity is needed. Existing resources such as the Immunization Handbook for Health Workers [22] and Rapid Immunization Skills Enhancement modules [47] may be leveraged, and novel resources may be developed, along with appropriate content delivery mechanisms. The existing supportive supervision mechanism [48] may be leveraged for performance tracking of supervisors and identifying candidates for reward and recognition. For block- and district-level officials who manage the RI workstream, a leadership-cum-technical skilling academy may be established to improve program governance capacity. On-the-job mentorship of ASHAs and ANMs may be explored through ASHA Facilitators and ANM Supervisors, respectively. During the COVID-19 pandemic, live online training and dissemination of prerecorded videos were attempted; these videos could be replicated in the future, not only for immunization but also for other government programs [49]. Recently, the Office of State Immunization Officer with support from development agencies has been proactively identifying health worker capacity-building needs and has been undertaking rapid training campaigns, especially for newly appointed ANMs [50].

Additionally, ASHAs can be provided with repeated training to improve their capacity related to the immunization program, with a focus on understanding data, storing records, and documenting, which can lead to stronger program performance [51].

Furthermore, improving the motivation of health workers involved in immunization programs through a rewards and recognition initiative can help improve their performance and program outcomes [52].

### Strengthening program review systems and governance

With a view to transforming the immunization programme in the state into a highly effective and resource-optimized programme, initiatives should aim to promote decentralized budget planning and formulate targeted solutions to meet the needs of the local population. This could be supplemented by active district- and subdistrict-level reviews and budgeting mechanisms that ensure that resources are being deployed for high-impact initiatives. Key areas for improvement in program review mechanisms include conducting regular review meetings with structured agendas, using data and evidence to make decisions, and then following up on those decisions [53]. Accurate enumeration and validation of beneficiaries through headcount surveys and easy-to-use tools to track left-outs and dropouts can help the state reach and sustain 90% FIC. [52]

Since the development and dissemination of the roadmap, block, and district weekly review meetings, which were severely impacted during the COVID-19 pandemic, have received focus from stakeholders and are being held regularly. Subsequently, a fresh headcount survey was conducted state-wide, and the microplans were updated till the SHC level. Further, the government digitized these microplans to improve the visibility of planned sessions and inform supportive supervision.

### Immunization service delivery

Addressing in- and out-migration across districts, its impact on healthcare worker workload, competency, and documentation skills in ASHAs and embracing hyperlocal strategies are the chief components that emerged in the present study as areas for strengthening immunization service delivery in Bihar. Ensuring that the migrant population has equitable access to immunization services through innovative service delivery models (such as mobile health teams), beneficiary tracking and tracing mechanisms, and participatory community engagement/education strategies are some of the potential means to address this issue as well as its impact on ASHA workload [51, 54]

Due to a decline in the sessions held against plan, some beneficiaries may have to travel longer or less frequently to access immunization services, which presents a potential risk of them not completing the recommended immunization course or receiving delayed vaccination, that has a linkage with increased risk of infection among children and avoid epidemics and outbreaks [55].

Furthermore, in flood-prone districts specifically, local, and contextualized strategies are being developed and deployed by stakeholders. Conducting need-based catchup sessions on elevated grounds that are partially submerged is an example of tackling service delivery challenges during floods in the state. Such practices may be explored, contextualized, and leveraged in various scenarios to improve immunization service delivery. During the COVID-19 vaccination campaign in Bihar, to accelerate the pace of vaccination, geographies affected by floods saw health workers commute using boats (*Teeka Wali Naav* -Vaccination on Boat in Muzaffarpur) [56]. The use of boats to enable service delivery in areas with poor access is an existing concept in Bihar [57]; hence, as a viable input for routine immunization program, it may be continued.

### Vaccine supply, storage, and logistics

A well-functioning iSC ensures access to vaccines for the recipients in an equitable and timely manner at the last mile. The recommended interventions are divided into two categories: first, ensuring context-specific expansion of cold chain point network, multiyear cold chain space sufficiency and equitable distribution at the last mile, need-based CCE allocation/reallocation, and deployment of alternate CCEs (solar powered, long-range passive devices, etc.) in challenging geographies [58, 59]. Second, continuous improvement in iSC through quarterly cold chain performance review using the eVIN and NCCMIS datasets should be ensured, in accordance with the improvement plan developed as part of the State Effective Vaccine Management (EVM) assessment 2019–20 [60], effective planned preventive maintenance guidelines for CCE, tracking of supervisory visits to cold chain points, increasing the number of in-position cold chain technicians to ensure adequate workload distribution, supporting VCCHs in managing competing priorities and vaccine wastage monitoring.

### Immunization data systems

The availability of comprehensive and robust immunization data systems, as well as their utilization by health workers and program managers, are critical for coverage monitoring, programmatic efficiency, and active decision making. The recommended interventions are classified into two categories. First, complete digitization of immunization reporting [61], effective deployment of mobile data tools for accurate data collection [62], and integration of multiple immunization data systems into a single decision-making platform for quick action may help with the data availability aspect [63]. Leveraging learning from the CoWIN platform, the Government of India launched the U-WIN in 2023 to effectively digitize UIP [64]. A single source of information on immunization services, updated vaccination status, delivery outcome, and planning of RI sessions and documentation, U-WIN is expected to address and tackle challenges with manual record keeping. For strengthening immunization data systems, streamlined implementation and utilization of U-WIN will be critical [65, 66].

Second, rapid data validation exercises [67] through specialized external teams, targeted capacity building of health workers [51], data-based training of district and block-level programme managers, and stronger data recording and reporting processes may help with the data quality aspect. The development of electronic decision-support tools that provide managers with on-demand analytics would complement these actions and enable data-driven decision making [68].

### Demand for immunization services

With immunization services generally available and accessible to most beneficiaries in Bihar, effective demand-side processes for immunization services are critical for the next phase of growth. Once institutionalized, demand generation initiatives can refocus the immunization programme's focus from traditionally being 'push' based to being 'pull' driven, with increased awareness in the population about the need for and importance of vaccines as well as more active public participation in ensuring a consistently high level of immunization coverage in their respective communities. It is recommended that the state builds the capacities of community leaders to engage in nontraditional mobilizing techniques to instill community ownership in promote vaccine demand [69]. There is a need to expand the level of caregiver knowledge regarding the specifics of the immunization schedule, session location/timings and management of postvaccination effects. New-age interventions involving technologies, particular those which help citizen engage directly with the health system and enable them to become active proponents of the program while reducing the dependence on ASHAs are needed [70, 71]. Leveraging the existing touchpoints in the mother‒child health life cycle to deliver interpersonal communications in favor of immunization is also recommended [72].

## The roadmap for improving routine immunization coverage in Bihar

**Fig. 6:**
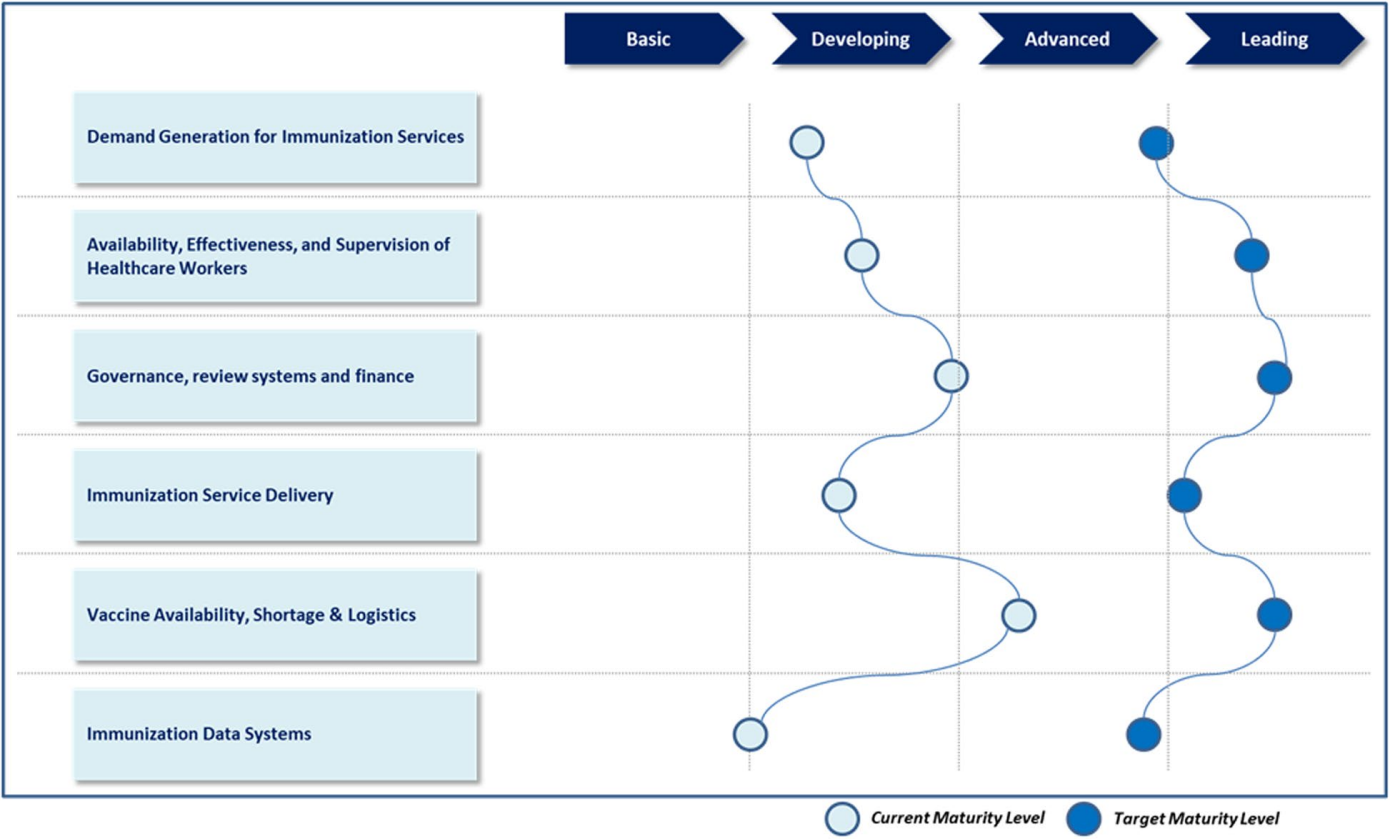
4-point maturity scale with current and target maturity levels

The roadmap developed through the four-phase process included a bouquet of interventions summarized under the identified thematic areas. The interventions adhered to a set of guiding principles that included decentralized decision-making, localized deployment of contextual solutions, and a focus on demand-promotion strategies. The identified program components/subcomponents were each assigned an index on the 4-point maturity scale (refer Fig. 6), which was used to assess the current and envisage the target maturity levels for the program component, considering the timeline of the roadmap and the feasibility of the intervention implementation. An additional excel file displays the maturity indexes assigned to each immunization program component in more detail (see Additional file 1).

**Fig. 7:**
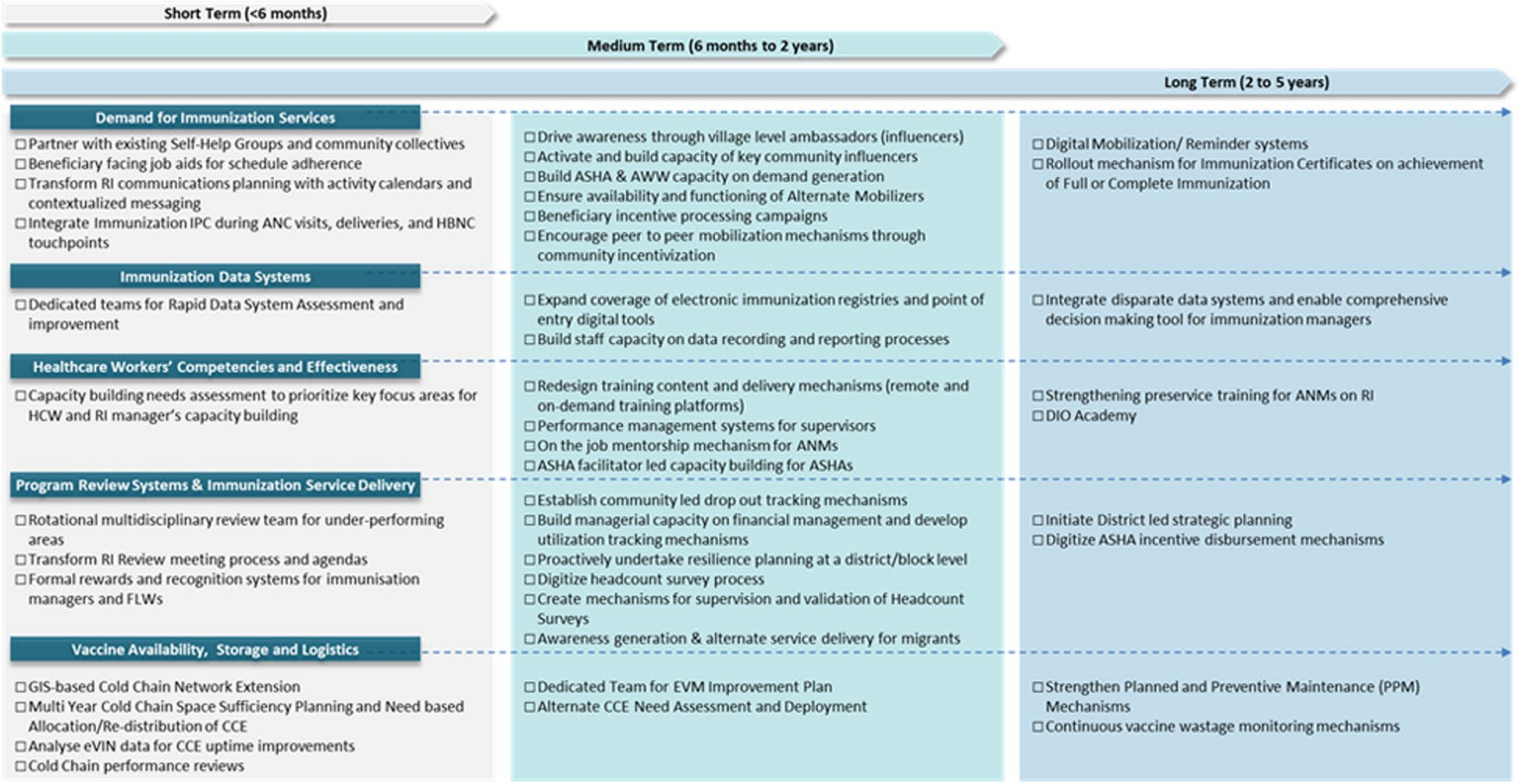
Prioritized list of interventions in the routine immunization roadmap for Bihar

The interventions identified through the outlined process were staggered into short-term, medium-term, and long-term interventions to help GoB breakdown the strategic goal of achieving and sustaining 90% FIC into manageable steps and allocate resources effectively. In terms of allocation across each term, short-term interventions may require more immediate and tangible resources, while medium- and long-term interventions may require more strategic planning and resource allocation. Considering the overall timeframe used to construct the roadmap, the short-term duration was 6 months, the medium-term duration was 2 years, and the long-term duration was 5 years. Figure 7 outlines the key interventions recommended as part of the roadmap.

## Limitations

Qualitative research by its nature is subject to biases, perceptions, and asymmetric or incomplete information from the research subjects, and while the use of semi-structured questionnaires mitigates this to some extent, the overall limitation of subjective opinions of participants applies here.

The initial plan to conduct more extensive field visits across the state was disrupted by travel restrictions due to the pandemic context and the availability of interviewees in the districts visited. This was managed by reverting to virtual and digital mechanisms to connect with the earmarked interviewee; in nine out of fourteen districts included in the study, virtual interviews were conducted to supplement the findings collected from the other districts.

To ensure the generalizability of the findings, the diversity of interviewees in terms of both geography and function was included by design—the research team interviewed stakeholders across multiple cadres and organizations across fourteen districts out of thirty-eight in Bihar. The districts were selected purposively, and potential selection bias may have crept in as a result; however the selection of districts that represent both ends of the immunization performance spectrum was included as a possible mitigation strategy, and additionally it was ensured that districts which faced unique circumstances were included—such as those with challenges related to floods, access challenges due to internal security challenges, urban areas, high instances of vaccine hesitancy, districts with high interstate migration, and districts with international migration to neighboring countries of Nepal and Bangladesh were all represented.

Given that the opinions shared by interviewees are indicative of the specific experiences and circumstances of the interviewees and possibly applicable to specific geographical areas, health facilities, villages, communities, or cadres, care was taken to caveat them by presenting them as opinions of the respective interviewee(s). When a multitude of divergent opinions or responses were offered by interviewers, it was ensured that both sets of views were represented.

Deductive coding of qualitative responses has distinct advantages in ensuring that consistency of analysis with previous research is maintained; however, the challenge of researcher-induced biases in predefining the codes may be present.

## Conclusion

The State of Bihar is among the first in India to develop and launch a multiyear roadmap to improve immunization coverage. The key contribution of the researchers here is to conduct a structured diagnosis of the immunization system and recommend a set of interventions that would support the government to reach and maintain 90% full immunization coverage. The lessons learned from Bihar’s Immunization Roadmap development project can provide insight for other states and countries that aim to formulate similar action plans.

### Supplementary Information

Supplementary Material 1.

Supplementary Material 2.

## Data Availability

The datasets generated and/or analyzed during the current study are not publicly available given the nature of the datasets but are available from the corresponding author on reasonable request.

## Abbreviations

AEFI: Adverse Effect Following Immunization
ANM: Auxilliary Nurse Midwife
APHC: Additional Primary Health Center
ASHA: Accredited Social Health Activist
AVD: Alternate Vaccine Delivery
AWW: Anganwadi Worker
BAM: Block Accounts Manager
BCM: Block Community Manager
BHM: Block Health Manager
BMGF: Bill and Melinda Gates Foundation
BWR: Block Weekly Review
CCE: Cold Chain Equipment
CCP: Cold Chain Point
CCT: Cold Chain Technician
CES: Coverage Evaluation Survey
CHC: Community Health Center
DEO: Data Entry Operator
DIO: District Immunization Officer
DTFI: District Task Force for Immunization
DVS: District Vaccine Store
DWR: District Weekly Review
EVM: Effective Vaccine Management
GAVI: Global Alliance for Vaccines and Immunisation
GNM: General Nurse Midwife
HMIS: Health Management Information System
ICDS: Integrated Child Development Services
IMI: Intensified Mission Indradhanush
IMR: Infant Mortality Rate
IPHS: Indian Public Health Standards
MMR: Maternal Mortality Rate
MO: Medical Officer
MOIC: Medical Officer Incharge
MPR: Monthly Progress Report
NCCMIS: National Cold Chain Management Information System
NFHS: National Family Health Survey
NHM: National Health Mission
PHC: Primary Health Center
PIP: Program Implementation Plan
RCH: Reproductive and Child Health
RHS: Rural Health Statistics
ROP: Record of Proceedings
RVS: Regional Vaccine Store
SDH: Sub District Hospital
SEPIO: State Expanded Programme on Immunization Officer
SHC: Sub Health Center
SRS: Sample Registration System
STFI: State Task Force for Immunization
SVS: State Vaccine Store
UNICEF: United Nations International Children's Emergency Fund
UIP: Universal Immunization Program
UPHC: Urban Primary Health Center
VCCH: Vaccine Cold Chain Handler
WHO: World Health Organisation
