# Supplementary material for: Developing a roadmap to reach and sustain 90% full immunization coverage through a cross-sectoral system strengthening strategy in Bihar, India
Source: BMC Health Serv Res. 2024 Aug 14;24:933. doi: 10.1186/s12913-024-11380-7 (PMC11325628; doi:10.1186/s12913-024-11380-7)
Supplement: Supplementary file 2 — Supplementary Material 2. [file 12913_2024_11380_MOESM2_ESM.docx]

# **Interview Questionnaires**

## **Questions to be asked from DIOs (and other District level officials)**

*Note: Similarly worded questions to be asked from the state level officials as well*

1. What is the immunization coverage of the district? How has it evolved over the years?
2. What are the characteristics of the district?
   1. Rural or Urban?
   2. Floods?
   3. Underprivileged communities?
   4. Vaccine Hesitant areas?
   5. Migration?
3. What are the key challenges to reaching 90% FIC in this district?
4. Is demand for immunization services a challenge in the district?
   1. Are there identified regions with demand challenges?
   2. What are the key demand side challenges?
      1. Lack of IEC?
      2. Vaccine Hesitancy?
      3. Migration ?
   3. What methods are deployed for demand generation?
      1. Are the staff adequately trained for creating immunization demand?
      2. In addition to the existing methods, is there any scope for involving KOLs/Jeevika groups in creating demand?
      3. Scope for targeted demand generation methods?
5. What are the key modes of program’s review mechanism?
   1. How frequently are reviews done? What is the agenda of these reviews?
   2. Who are the reviewers?
   3. What data systems are used for reviewing?
   4. Are action plans created and tracked subsequently?
   5. What is the involvement of MO/ICs and MOs in program reviews?
6. Do the supportive supervision activities function well? Which cadre has the primary responsibility for supervision of sessions? How can increased supervision visits by government cadres be ensured?
   1. Is sufficient supervisory cadre present? What can be done to ensure adequacy?
7. How does the relative lack of health infrastructure affect immunization services?
   1. Does it impact service delivery for HRAs?
   2. Does it overburden the ANMs conducting outreach (greater distances)?
8. Is the HCW staff appropriately distributed?
   1. If not, what drives the inequities across regions? What can be done to reduce this?
   2. Are ANMs difficult to identify, train, and hire in such places?
9. Which development partners are working here, and what are the areas of their support?
10. What are the key challenges in healthworker’s capacity which impede the immunization program?
    1. What are the different trainings available for different HCW cadres?
       1. What training methods are deployed?
       2. Are trainings tracked and improvement areas followed up on?
    2. Are and trainers well capacitated to deliver quality RI trainings?
11. Are there challenges in ensuring that H2H surveys capture the complete population, especially migrants?
    1. Is there any scope for digitization of H2H surveys in Bihar?
    2. Are there challenges in due list creation?
    3. Is Supervision of H2H survey process done?
12. How is drop out tracking done? How is it monitored?
13. What are the key challenges in microplanning?
    1. How frequently are the plans revised?
    2. How is equity of services ensured? How is planning for HRA or HTR populations done?
    3. Are some areas missed?
    4. Is real time data available?
14. Data Systems
    1. What are the key drivers for poor accuracy and reliability of HMIS data?
    2. How is MPR/Tally Sheet data reviewed for accuracy and completeness?
    3. What is the plan for scaling of RCH/ANMOL?
    4. What is the quality of trainings provided on data systems?
15. What are the key challenges that are faced as a part of the DIO’s role?
16. How does the budgeting process for NHM-PIP work at a district level? What are the challenges?
    1. Are there tracking mechanisms in place?
    2. What are the key challenges in ensuring efficiency in disbursement processes?

## **Questions to be asked from Medical Officer Incharge (and other Block level officials)**

1. What is the immunization coverage of the block? How has it evolved over the years?
2. What are the characteristics of the block?
3. Rural or Urban?
4. Floods?
5. Underprivileged communities?
6. Vaccine Hesitant areas?
7. Migration?
8. What are the key challenges to reaching 90% FIC in this block?
9. Is demand for immunization services a challenge in the block?
10. Are there identified regions with demand challenges?
11. What are the key demand side challenges?
12. Lack of IEC?
13. Vaccine Hesitancy?
14. Migration?
15. What methods are deployed for demand generation?
16. Are the staff adequately trained for creating immunization demand?
17. In addition to the existing methods, is there a scope for involving KOLs/ Jeevika groups in creating demand?
18. Scope for targeted demand generation methods?
19. What are the key modes of program reviews?
20. How frequently are reviews done? What is the agenda of these reviews?
21. Who are the reviewers?
22. What data systems are used for reviewing?
23. Are action plans created and tracked subsequently?
24. What is the level of involvement of MO/ICs and MOs in program reviews?
25. Infrastructural Challenges
26. Does it impact service delivery for HRAs?
27. Does it overburden the ANMs conducting outreach (greater distances)?
28. Why is setting up new facilities difficult?
29. Buildings
30. HR
31. Electricity
32. Do the supportive supervision activities function well? Which cadre has the primary responsibility for supervision of sessions? How can increased supervision visits by government cadres be ensured?
33. Is sufficient supervisory cadre present?
34. What can be done to ensure adequacy?
35. HCW Adequacy ad Distribution
36. If not, what drives the inequities across regions? What can be done to reduce this?
37. What is the process for hiring ANMs and other staff?
38. Why are supervisory cadres relatively poorly staffed?
39. Are ANMs/LHVs difficult to identify, train, and hire in such places?
40. Microplanning
41. Whether the micro-planning exercise was conducted for all the ANMs
42. How many ANMs submitted the updated micro planning formats to the block
43. How frequently are the plans revised?
44. How is equity of services ensured? How is planning for HRA or HTR populations done?
45. Are some areas missed?
46. Which development partners are working here, and what are the areas of their support?
47. Priorities: What are the three key priorities for the next three years for RI Improvements in this block?
48. How does the budgeting process for NHM-PIP work at a district level? What are the challenges?
49. Are there tracking mechanisms in place?
50. What are the key challenges in ensuring efficiency in disbursement processes?

## **Questions to be asked at a Session Site (from an ANM/GNM/ASHA Facilitator/ Caregivers)**

1. Microplanning and Immunization Journey of caregivers:
   1. How is the microplan developed and what is the typical journey like for the caregivers?
   2. What is the range of beneficiaries visiting the session sites?
   3. Are all localities covered as per the microplan? Are there any missed area?
   4. Has the microplan changed/ not changed in the last three year?
   5. How is it decided that a new session site needs to be opened?
2. Headcount Survey:
   1. How is the headcount survey done? What are challenges faced?
   2. Are all households generally found in the H2H survey? What are some areas of improvement in the survey process?
3. Demand Generation Activities
   1. Does the ANM/ASHA collaborate with KOLs/ Jeevika groups
   2. Is the ANM involved in demand generation?
   3. What does the ANM do to convince hesitant families
   4. Communications plan
4. Data records:
   1. What is the process of developing duelist?
   2. What is the quality of duelist developed? Is there any discrepancy between H2H survey, due list, Tally Sheet and HMIS-Monthly Progress Report?
   3. Is the Tally sheet, RCH and HMIS-MPR (and relevant data records) present at the session sites? Are they updated and have logical consistencies?
   4. What are the challenges with the present data recording systems?
5. Vaccine Logistics
   1. How is the vaccine delivery done? What are the reverse logistics modalities? Are there any challenges associated with it?
6. Mobilization
   1. What is process of mobilizing the caregivers/beneficiaries for earmarked health services? Who is accountable for doing it?
7. AAA Meetings:
   1. What is generally discussed in this meeting?
   2. Who are the committee members?
   3. What action is taken?
   4. How frequently is the meeting done?
   5. Any there any records to refer?
8. Capacity Building:
   1. What are the areas where ANM requires more training?
   2. What is the quality of traditional trainings as per Govt’s plan of action?
9. Related to ASHAs:
   1. Are there any awareness challenges related to the ASHA incentives?
   2. What are the other challenges associated with the ASHAs?
10. Dropout Tracking process:
    1. What methods are adopted for drop out tracking? Are these methods being followed?
11. Supportive Supervision:
    1. How frequently is it supervision done? Who is supposed to do it?
    2. Is action taken basis the supervisory visit findings? How can supervision aspects for immunization program be strengthened?
12. Migrants
    1. What is the general person of migrants visiting the session site? How are they being delivered the services?
    2. Any additional aspects to consider or implement when managing migrant beneficiaries?
13. Program Reviews
    1. How are program reviews conducted? What is generally discussed? What happens to the decision points, actionables and their followup?
    2. Are the challenges generally addressed? What else should be considered for strengthening program reviews?

## **Questions to be asked from ASHAs and AWWs**

1. Who is the decision-making authority for child immunisation in the households in this village?
   - Mother
   - Father
   - Others (please mention)
2. Is a special method followed to mobilise migrant families? (Please ask questions based on specific topography/demography of the block – floods, vulnerable groups etc.)
3. Are there certain communities in this area which are challenging to mobilise? If yes, then how are they convinced to bring their children for immunization?
4. Are there or have there been any misinformation or rumours spread in the community about vaccines or syringes?
5. How are ASHAs/AWWs trained or instructed on health mobilisation? Are they given an initial set of recommendations/instructions?
6. Who are caregivers most likely to listen to or take advice from regarding healthcare information? (ASHA, ANM, AWW or other)
7. Are there any specific reasons for vaccine hesitancy that exist in the community?
8. What are the different types of data recording and reporting that are required to be complied? What are the benefits of these to ASHAs/AWWs’ work? What are the key challenges faced?
9. (ASHA Incentives) Which health service takes up the most time for ASHAs/AWWs to provide counselling on? Can the ASHA incentive process of immunization be improved in any way?
10. (Review Meetings) What are the different review meetings that ASHAs are required to attend in a month? Are they useful and/or how can they be improved?

## **Questions to be asked from Caregivers (Mothers/Fathers/etc.)**

1. Are you aware of the vaccines being given and what they protect the child from?
2. What are various challenges that you face to reach and accept the vaccines?
3. What existing aspects of the immunization program are of good quality in your opinion?
4. What new aspects would you like to see in future for helping with safe & timely vaccination?

## **Questions to be asked from a Community Leaders/Members**

1. Do you know that new-born children and pregnant women must be vaccinated? Are you aware of the immunization program run by government? (2 min)
2. Bihar has an overall coverage of ~80%. Some beneficiaries still miss the vaccines. What are the various challenges that exist in the community towards accessing immunization? (5 min)
3. What existing aspects of the immunization program are of good quality in your opinion? (5 min)
4. What new aspects would you like to see in future for helping with safe & timely vaccination? (5 min)
5. How can we improve demand generation aspect of the immunization, so that beneficiaries start demanding for vaccination in addition to the health-workers’ effort? (5 min)
6. Have you attempted any intervention/ initiative to improve the health services/ immunization? Please tell us about it. (5 min)
7. What additional role would you be willing play to improve the immunization services (demand improvement, community mobilization, advocacy for safety/importance of vaccines, convincing resistant households, etc.)? (5 min)
8. What support from government would you find helpful in order to take up these activities? (5 min)

## **Questions to be asked from development agencies**

**Immunization Coverage and Equity Outcomes**

- What key factors explain Bihar’s relative success in improving FIC and IMR rates over the past decade?
  - Bihar has made improvements to both child health and maternal health outcomes despite challenges related to health infrastructure, floods, literacy, poverty. How has Bihar been able to overcome these challenges, what strategies have enabled Bihar to make these gains?
- What are the key barriers to reaching 90% FIC in Bihar?
  - Which factors are responsible for stagnation in gains in FIC over the past four years?
  - What are the key areas that the government and partners would have to focus on in order to achieve this target?
- There are observed inequities in both Immunization and other child health outcomes across population sub-groups. What factors and mechanisms explain the differences in FIC and other child health outcomes across divides of gender, caste, religion, and geographies?
  - Why do the northern districts of Bihar consistently perform poorly across health indicators? Is this primarily explained by the accessibility challenges due to floods?
    - What local innovations have been adopted in immunization service delivery in the flood prone districts to overcome the accessibility challenges?
  - Do conditional cash transfers/ incentives targeting specific sub groups (such as Mukhyamantri Kanya Utthan Yojana) work in addressing inequity issues?

**Program Management**

- What are the key challenges in program review and monitoring?
  - What are the different mechanisms for program review and monitoring across state/district/block levels?
  - What modes of reviews have been adopted?
- Do the supportive supervision activities function well?
- What is your view on partnering with institutions like medical colleges, IITs etc. to lead some aspects of the immunization program in the state?

**Partners**

- Presence of partners across components of immunization and RMNCHA+ is high in Bihar. In your view, what advantages or challenges does this bring about?
  - How do you foresee the roles of partners changing over the coming years?
- The WHO and UNICEF cadres (SMOs, FMs SMNet) are being transitioned. What are the key challenges in ensuring a successful transition of these cadres?

**Health and Immunization Infrastructure**

- There are variations across districts in population dependence on facilities such as SHCs, APHCs, PHCs, with all districts comparing unfavourably against IPHS norms. The dependence is worse in Northern Bihar Districts. Is this a conscious strategy to focus on outreach rather than augmenting infrastructure?
  - Does high population burden lead to poor quality of care for beneficiaries?
  - Does it affect demand for immunization services?
  - Do MICs signal a shift in state strategy to greater focus on immunization at fixed sites?
  - Urban infrastructure is particularly poor, what could be the reasons for this?
    - Does it impact immunization coverage in urban areas?

**Healthcare Worker availability**

- While the state has high ANM availability overall, there are wide variations across districts?
  - Are there challenges in identifying and recruiting ANMs in northern districts?
    - Within districts, are there challenges in recruiting ANMs from certain rural areas and communities?
  - Supervisor availability is low, with LHV/Health Assistants severely understaffed. What are the challenges in ensuring availability of supervisory cadres?
  - Does the supervisory mechanism (Supportive Supervision) work well? What are the key challenges?

**Healthcare Worker Capacity**

- What are the key challenges in HCW capacity which impede the immunization program?
- What are the different trainings available for different HCW cadres?
  - What training methods are deployed?
  - Are trainings tracked and improvement areas followed up on?
- Are training institutes and trainers well capacitated to deliver quality RI trainings?
- What are the reasons for low budgetary plans for trainings?

**Session Strategy**

- The overall number of immunization sessions have remained the same (and even slightly decreased in some years), despite the addition of ANMs in recent years. Is this a conscious strategy by the state to ensure high turnouts?
  - Is a need for more number of immunization sessions felt?
  - Can increasing the availability of services still contribute to increasing FIC from 80% to 90%
  - In your opinion, is the state well placed to increase the number of immunization sessions?

**Headcount Surveys & Due Lists**

- Are there challenges in ensuring that H2H surveys capture the complete population?
- Do you see a scope for digitization of H2H surveys in Bihar?
- Are there challenges in due list creation?

**Microplanning**

- What are the key challenges in microplanning?
  - Are the microplanning methods presently deployed sufficient to capture the last 20%?
  - Is there a need to redefine the HRA population criteria?

**Demand**

- Is demand for immunization services a key challenge in Bihar?
  - Do demand side challenges disproportionately affect certain population sub groups?
- What community norms and practices prevent beneficiaries from availing health and immunization services in spite of availability?
- What interventions have worked in increasing healthcare demand which may be applicable for Bihar Immunization?

**Data Systems**

- What are the key challenges in quality and usage of data systems such as HMIS, RCH?
- What challenges have been observed in scaling the RCH Portal?
  - What can be done to increase the uptake?

**Budgeting and Financing**

- How well do the budgeting and planning processes for immunization work?
- Is Bihar's immunization program adequately financed?
- What explains the low budgetary utilization across items?
  - Are there tracking mechanisms in place?
  - What are the key challenges in ensuring efficiency in disbursement processes?
